# Effectiveness of Contralaterally Controlled Functional Electrical Stimulation versus Neuromuscular Electrical Stimulation on Upper Limb Motor Functional Recovery in Subacute Stroke Patients: A Randomized Controlled Trial

**DOI:** 10.1155/2021/1987662

**Published:** 2021-12-22

**Authors:** Songhua Huang, Peile Liu, Yinglun Chen, Beiyao Gao, Yingying Li, Chan Chen, Yulong Bai

**Affiliations:** ^1^Department of Rehabilitation Medicine, Huashan Hospital, Fudan University, Shanghai 20000, China; ^2^Department of Rehabilitation Medicine, Huashan Hospital North, Fudan University, Shanghai 20000, China; ^3^National Center for Neurological Disorder, Shanghai, China

## Abstract

**Purpose:**

To compare the effectiveness of contralaterally controlled functional electrical stimulation (CCFES) versus neuromuscular electrical stimulation (NMES) on motor recovery of the upper limb in subacute stroke patients.

**Materials and Methods:**

Fifty patients within six months poststroke were randomly assigned to the CCFES group (*n* = 25) and the NMES group (*n* = 25). Both groups underwent routine rehabilitation plus 20-minute stimulation on wrist extensors per day, five days a week, for 3 weeks. Fugl-Meyer Assessment of upper extremity (FMA-UE), action research arm test (ARAT), Barthel Index (BI), and surface electromyography (sEMG) were assessed at baseline and end of intervention.

**Results:**

After a 3-week intervention, FMA-UE and BI increased in both groups (*p* < 0.05). ARAT increased significantly only in the CCFES group (*p* < 0.05). The changes of FMA-UE, ARAT, and BI in the CCFES group were not greater than those in the NMES group. The improvement in sEMG response of extensor carpi radialis by CCFES was greater than that by NMES (*p* = 0.026). The cocontraction ratio (CCR) of flexor carpi radialis did not decrease in both groups.

**Conclusions:**

CCFES improved upper limb motor function, but did not show better treatment effect than NMES. CCFES significantly enhanced the sEMG response of paretic extensor carpi radialis compared with NMES, but did not decrease the cocontraction of antagonist.

## 1. Introduction

Neuromuscular electrical stimulation (NMES) is a modality widely used in stroke rehabilitation for motor impairment by improving or assisting volitional movement [1]. Cyclic NMES offers a preset and passive stimulation on specific muscles with on-off cycles of repetitive mode. The therapeutic effect of NMES for upper limb motor impairment after stroke has been shown in several randomized controlled trials [2]. The repetitive movement training induced by NMES may facilitate motor relearning. However, goal-oriented active repetitive training is not easy to carry out by cyclic NMES in acute or subacute phase after stroke, especially in severe cases. As a kind of physical therapy, NMES consists in evoking contractions by applying an electrical current over the muscle via surface electrodes. It represents an incomplete activation of the neuromuscular system, and the use of submaximal training intensities should partly account for its lower efficiency on muscle strength than resistance training [1]. Some studies found more significant functional improvements by cyclic NMES when paired with simultaneous voluntary effort using residual movement [2].

Contralaterally controlled functional electrical stimulation (CCFES) is a unique stimulation modality using a motor signal detected from the volitional movement of the nonparetic limb to control the electrical stimulation delivered to the paretic limb, which induces similar movement on the paretic limb [3]. Different with cyclic NMES, CCFES enables active participation of both sides of limb as well as self-control of the timing and intensity of stimulation to the paretic limb, without requirement of residual movement. A small clinical study has reported that CCFES improves hand dexterity in subacute stroke patients [4]. The effect of CCFES on wrist extension (active range of motion) and the improvement of the upper limb function were reported in stroke patients in 3 months and 15 days [5, 6]. The changes of Motricity Index of the upper limb and strength of extensor carpi assessed by the manual muscle power test were also reported. But the Motricity Index was not significantly enhanced along with the improvement of active range of motor of wrist extension. Surface electromyography (sEMG) evaluation may be an appropriate assessment of muscle activation [7, 8].

This study is aimed at comparing the effectiveness of CCFES versus NMES on upper limb function recovery in 6 months poststroke by upper limb and hand functional assessment and surface electromyography (sEMG) evaluation. We hypothesized that CCFES had better effect on the upper limb function and muscle activation than NMES in subacute stroke patients.

## 2. Materials and Methods

This study was designed as a parallel randomized controlled trial. Doctors who evaluated the outcome assessments were blinded to the allocation. The study protocol was approved by the ethics committee of Huashan Hospital, Fudan University (the approval number 2019-006). This study has been registered with Chinese Clinical Trial Registry (http://www.chictr.org.cn/) (No. ChiCTR1900021770).

### 2.1. Subjects

Patients admitted to the Department of Rehabilitation Medicine, Huashan Hospital North, Fudan University, from March 2019 to March 2020 were recruited. All patients were given informed consent for this study, and written consents were provided by the patients or their legally authorized representatives.

Inclusion criteria were as follows: (1) diagnosis of a first-ever stroke confirmed by head CT or MRI scanning, (2) well general condition with stabilized vital signs and normal consciousness, (3) age 30~85 years, (4) Brunnstrom recovery stage one to four for the affected upper limb, (5) 7 days to 6 months after stroke onset [9], (6) unilateral lesion indicated by CT or MRI, and (7) voluntary for this study with a signed informed consent.

Exclusion criteria were as follows: (1) reversible stroke; (2) severe visceral organ (e.g., heart, lung, liver, and kidney dysfunction); (3) severe cognitive dysfunction, MMSE < 23; (4) with a history of mental disease and cannot cooperate in rehabilitation treatment; (5) deaf-mutes; (6) unable to receive treatment in designated hospital at specific time or unable to be followed up regularly; (7) implanted with cardiac pacemaker; and (8) with upper limb dysfunction due to other causes.

The administrative assistant of the study who did not participate in the treatment and assessment assigned the patients to either the NMES group or the CCFES group using random number table generated by computer and allocated 1 : 1 by concealed sequentially numbered envelopes.

### 2.2. Study Protocol

Both groups went through routine rehabilitation (1 hour/day) for 5 days per week over a period of 3 weeks, including posture management (sitting, standing, and sit to stand), abnormal reflex inhibition, proprioceptive neuromuscular facilitation, and occupational therapy. Routine rehabilitation was performed by therapists blinded to group allocation. Both CCFES and NMES were provided in addition to routine rehabilitation training. Accompanying diseases (e.g., hypertension, coronary artery disease, and diabetes) were treated with medicines.

In the CCFES group, contralaterally controlled functional electrical stimulator (DC-L-500, Jiangsu NeuCognic Medical Co., Ltd., Jiangsu, China) was used to stimulate wrist extensors of the paretic side controlled by the nonparetic side. Subjects sit with arm and hand resting at side and forearm pronated. The 2 stimulating electrodes were placed on the muscle belly of extensor carpi radialis of the paretic side. The main controller for detecting nonparetic wrist extension and triggering stimulation on paretic wrist extensors was worn on the back of the nonparetic hand. Before stimulation, subjects were asked to voluntarily extend the nonparetic wrist to certain angle according to the instruction (0, 45, and 20 degrees) and recorded by the main controller. The extension of the paretic wrist was elicited by the electrical stimulation from the main controller when it detected the motion of the nonparetic wrist (at least 20-degree extension). The stimulation is aimed at generating 20-25 degrees of wrist extension on the paretic side. The therapist would instruct the nonparetic wrist extension and adjust the stimulating intensity ensuring to elicit 20–25-degree wrist extension on the paretic side without causing pain or any discomfort (a sensory sustainable range). Subjects were instructed to relax the paretic arm during the treatment. The waveform of stimulation was biphasic rectangular wave with frequency of 35 pps and pulse width of 200 *μ*s. Subjects were asked to maintain the nonparetic wrist extension for 10 s so that the stimulation on the paretic side could last. Once the nonparetic wrist relaxed and went back to 0 degree, the stimulation ceased. The interval of every motion and stimulation was set as 10 s. The CCFES treatment was performed 20 min/session, 1 session/day, 5 consecutive days/week, for 3 weeks. A 5 min practice session was performed in the initial of the CCFES therapy to make sure the subjects know how to accomplish the treatments.

In the NMES group, the stimulation was conducted by the bio-feedback electrical stimulator (MyoNet-BOW, Shanghai Ncc Electronic Co., Ltd., Shanghai, China). Subjects sit with arm and hand resting at side and forearm pronated. The 2 stimulating electrodes were placed on the muscle belly of extensor carpi radialis on the paretic side. Subjects were instructed to relax the paretic arm during NMES. The waveform of stimulation was biphasic rectangular wave with frequency of 35 pps and pulse width of 200 *μ*s. The stimulation and relaxation time was set as 10 s : 10 s. The stimulation intensity was adjusted to the level of tetanic contraction, which would elicit 20-25 degrees of wrist extension of the paretic hand, without causing any pain sensation. The electrical stimulation treatments were performed 20 min/session, 1 session/day, 5 consecutive days/week, for 3 weeks.

### 2.3. Outcome Assessment

Functional evaluations were performed by two doctors blinded to group allocation at baseline and after a 3-week intervention.

The primary outcome was action research arm test (ARAT). This test is used to evaluate the motor performance of the arm and hand including 4 subscales (grasp, grip, pinch, and gross movement). The 19 items are rated on a 4-point scale scoring from 0 (no movement) to 3 (normally performed movement). The maximum score is 57. On the basis of clinical experience and estimates reported for similar outcome measures, the minimal clinically important difference (MCID) of ARAT was set at 10% of the total range, which was 5.7 points [10].

The secondary outcomes included the following:

1. Motor function of Fugl-Meyer Assessment of upper extremity (FMA-UE): the motor function of FMA-UE evaluates the tendon reflexes and the performance of given tasks involving the shoulder, elbow, wrist, and hand. Each item is rated on a 3-point scale scoring from 0 to 2, except for the reflex activity which has only 2 points, scoring 0 or 2. Scoring 0 means no reflex can be elicited or cannot do the given task. Scoring 1 means the task can be performed partially. Scoring 2 means the task can be performed fully. The maximum score of motor function of FMA-UE is 66
2. Barthel Index: 10 items, including feeding, fecal and urinary incontinence, dressing and undressing, grooming, toilet use, bathing, transfer (e.g., from chair to bed), walking, and climbing stairs. The total score is 100
3. Surface electromyography (sEMG): the surface electromyographic signals of the extensor carpi radialis and flexor carpi radialis on both side were recorded during active wrist extension by surface electromyography apparatus (MyoMove-EOW, Shanghai Ncc Electronic Co., Ltd., Shanghai, China). The signal was amplified and band pass filtered (5-500 Hz) prior to sampling. The subjects were trained before signal collection to understand the whole procedure. During the collection, the subjects were required to try their best to extend the wrist and maintain for about 3 s and then relax for 5 s, repeating for 3 times. The signals were recorded and generated automatically to the root mean square (RMS) values by the software installed with the surface electromyography apparatus. The RMS of paretic extensor carpi radialis was standardized by calculating sEMG signal ratio in percentage, a ratio of RMS of the paretic side/the nonparetic side. Cocontraction ratio (CCR) of paretic flexor carpi radialis was calculated by the ratio of RMS of flexor carpi radialis/(RMS of flexor carpi radialis + RMS of extensor carpi radialis) [8]. The smaller CCR of paretic wrist flexors indicates the better motor control of voluntary wrist extension

### 2.4. Statistical Analysis

For baseline demographic and clinical characteristic comparability, chi-square test was used for categorical variables and *t*-test was used for continuous variables. Two-sample *t*-test was used to compare the change of each assessment (ARAT, FMA-UE, Barthel Index, and RMS of extensor carpi and CCR of flexor carpi) from baseline to end of intervention between groups. Paired *t*-test was used for comparison of each assessment between baseline and end of intervention in each group. The significance level is set at 0.05. SPSS 18.0 was used for statistical analysis.

Sample size was estimated according to the MCID of ARAT. SD was 6.0. Sample size was calculated using two-sample estimation at a level of significance of 0.05 with 90% powers.

## 3. Results

**Figure 1:**
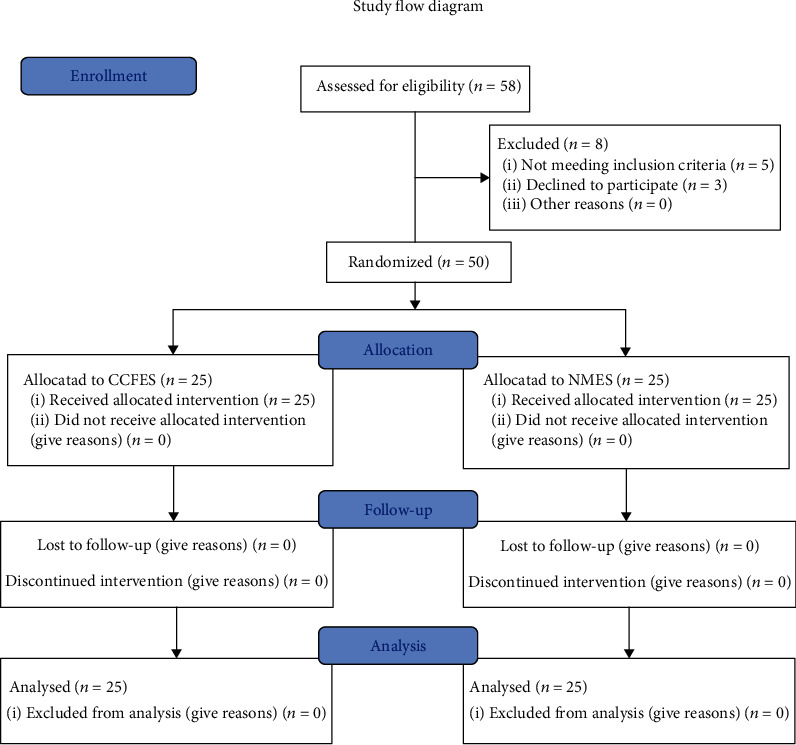
Study flow diagram. CCFES: contralaterally controlled functional electrical stimulation; NMES: neuromuscular electrical stimulation.

Fifty eligible patients were enrolled and randomly assigned into the NMES group (*n* = 25) and the CCFES group (*n* = 25) (Figure 1). There were no significant differences between groups in gender, type of stroke, side of affected hemisphere, and Brunnstrom recovery stage according to chi-square test (*p* > 0.05), nor in age and course of disease according to *t*-test (*p* > 0.05) (Table 1).

After a 3-week intervention, FMA-UE and Barthel Index increased significantly in both groups (*p* < 0.05). ARAT increased significantly only in the CCFES group (*p* < 0.05). The change of FMA-UE, ARAT, and Barthel Index from baseline in the CCFES group was not significantly greater than that in the NMES group. For sEMG evaluation, the improvement of RMS of extensor carpi radialis in the CCFES group was greater than that in the NMES group with the difference of 0.09 (95% CI 0.01-0.16, *p* = 0.026). NMES had no effect on RMS of extensor carpi radialis. The CCR of flexor carpi radialis in CCFES did not significantly decrease, and the change of CCR in CCFES was not significantly lower than that in the NMES group (Table 2).

No adverse events were reported during the intervention and follow-up in any of the groups.

## 4. Discussion

This randomized controlled trial compared the effectiveness of CCFES and NMES on upper limb motor function recovery in patients within 6 months poststroke. At the end of the 3-week intervention, the CCFES group gained greater improvement of RMS of extensor carpi radialis than the NMES group. Treatment effects of ARAT, FMA-UE, Barthel Index, and CCR of flexor carpi radialis were not significantly different between the CCFES and NMES groups. Both groups had greater scores of FMA-UE and BI at the end of intervention, while ARAT increased significantly only in the CCFES group.

CCFES improved upper limb function, but did not show greater treatment effect on assessed upper limb function (FMA-UE and ARAT) in our study. This finding was not consistent with reported studies. An early-phase study reported by Knutson et al. favored 6-week intervention of CCFES over NMES on improvement of FMA, box and block test (BBT), arm motor ability test (AMAT), and the angle of finger extension at the end of treatment and 1 month and 3 months posttreatment in subacute stroke patients [4]. However, the power of this study was limited by the small number of subjects. Later in 2016, Knutson et al. reported results in chronic stroke patients (>6 months) with longer treatment (12 weeks, 10 hours/week). The CCFES group had greater improvement on BBT performance than NMES at 6 months posttreatment but no significant gain on FMA and AMAT [11]. The stimulation duration and sessions were longer compared with our study. Besides, the stimulation was targeted on hand opening, along with functional task training assisted by CCFES, which may explain the greater gain in hand dexterity. In 2015, Shen et al. reported a study comparing the effectiveness of CCFES on wrist extensors versus NMES in patients within 3 months poststroke. After the 3-week intervention of CCFES, they reported greater improvement of FMA of upper extremity, the Hong Kong version of the Functional Test for the Hemiplegic Upper Extremity (FTHUE-HK), and Active Range of Motion (AROM) of wrist extension than NMES. The gain of FMA was 7.6 (SD1.4) in the CCFES group and 4.9 (SD1.4) in the NMES group, with a mean difference of 2.7 [5]. Our study reported a greater gain of FMA in the NMES group (6.04) and a smaller difference of gain between groups, with 1.76 (95% CI -2.65-6.17). Although our intervention protocol was similar to the study by Shen et al., the subject population might have different characteristics of central plasticity such as cortical excitability, interhemispheric inhibition, and integrity of the corticospinal tract. The central plasticity of subjects would be a confounder of the treatment effect of CCFES on functional assessment.

The recovery of muscle strength was also assessed in previous studies. Zheng et al. found that CCFES had better effect on wrist extensor strength than NMES in acute stroke patients [6]. Shen et al. reported no significant benefit for Motricity Index by CCFES in subacute stroke patients [5]. Our study used sEMG evaluation to measure the paretic muscle activation. The temporal bioelectrical signals of muscles recorded from the skin surface during muscle activation reflect the recruitment and synchronization of motor units. Although we set the stimulation in a “passive” mode, CCFES had greater treatment effect for extensor carpi radialis than NMES. The cocontraction ratio we used was defined as the proportion of cocontraction muscle forces to total muscle forces. Excessive cocontraction and abnormal muscle recruitment may limit the movement in the central nervous diseases. Studies have indicated higher cocontraction ratio of the affected limb in stroke [7] and cerebral palsy [8]. CCFES also favored decreased cocontraction of flexor carpi radialis, without significant difference with NMES. The results indicated that CCFES had a potential benefit for enhanced voluntary contraction of paralyzed muscle and muscular activation pattern. However, the improvement of muscular activation did not translate to greater functional gain in our study. We assumed longer intervention and combination of functional task training may enhance the translation.

NMES is one rehabilitation treatment option for stroke patients with motor function impairment. The motor learning mechanism is assumed to be the therapeutic effect NMES as it can induce repetitive movement. However, the effectiveness of NMES on function improvement has been questioned. Wilson et al. observed the effectiveness of 3 different modes of electrical stimulation (cyclic NMES, EMG-triggered NMES, and sensory stimulation without motor recruitment) on upper limb functional changes in stroke patients within six months poststroke. Yet they attributed the results to self-recovery rather than electrical therapies [12]. Combined with task-related training, functional electrical stimulation (FES) has been verified to promote motor recovery by changing the motor networks in the cortex and enhancing synaptic remodeling [13].

CCFES offers a new mode with combination of bilateral symmetrical motor training and self-controlled electrical stimulation. Bilateral symmetrical motor training is assumed to effectively activate the primary motor cortex (M1) ipsilateral to brain damages [14] and further rebuild new task-relevant neural networks based on residual neurons [15, 16]. This intervention may accelerate functional recovery through motor pathway facilitation [17, 18]. Cortical physiological studies conducted by transcranial magnetic stimulation showed that in the acute phase of stroke, patients' performance was limited to corticospinal damage [19]. While at 3 months poststroke or chronic phase, intracortical disinhibition may play a role in reorganization of cortical network and improvement of hand function [19–21]. Some transcallosal nerves that originated from the nonlesion side to lesion side show crucial effect on motor function recovery after neural disconnection [22]. In chronic stroke patients, bilateral arm training led to significantly higher increase in activation in the ipsilesional cortex (the precentral, the anterior cingulate, and the postcentral gyri and the supplementary motor area) and contralesional superior frontal gyrus. Activation change in the contralesional cortex was correlated with improvement in the Wolf Motor Function Test Time [23]. The theoretical model developed by Mudie and Matyas suggested that bilateral symmetric movement could promote interhemispheric disinhibition and allow the ipsilesional hemisphere to share a “template of motor network recruitment” from the contralesional hemisphere [16]. Coupled bilateral movement and EMG-triggered NMES on affected arm enhanced hand function and reaching task performance compared with NMES in chronic stroke [24, 25]. And compared with coupled bilateral movement and placebo stimulation, coupled bilateral movement and FES had better effect on improvement of functional test of upper limb and active range of wrist extension [26, 27]. A recent crossover study showed that CCFES (bilateral symmetric movement) reduced interhemispheric inhibition and maintained ipsilesional output when compared with NMES (unilateral-based therapy) [28]. Besides, the intention-to-control mode of stimulation offered by CCFES enables patients who have no or fewer residual motor function of affected limb to do functional-related repetitive training. By offering the illusion of good motor control to the patient, CCFES may integrate afferent sensory fibers to change the motor network in the cortex and enhance synaptic remodeling to facilitate brain plasticity.

There are several limitations to this study. The central plasticity of subject was not evaluated by electrophysiological or functional imaging investigation at baseline, which could be a confounder of the treatment effect of CCFES. And the effect of CCFES on central plasticity was not measured in this study. The duration of intervention was short, which may reduce the effect of CCFES. The lasting effect of possible central plasticity by CCFES was unable to be explored due to the lack of longer follow-up assessments. The study included patients with different Brunnstrom recovery stages of upper limb, which may lead to confusion as to the best application of CCFES. In the future study, adding a new intervention group of voluntary movement on nonparetic side (without CCFES)+NMES on paretic side as a bilateral movement training may provide more information.

## 5. Conclusions

CCFES improved upper limb motor function but did not show better therapeutic effect than NMES after the 3-week stimulation on extensor carpi radialis according to functional assessments in subacute stroke patients. CCFES enhanced the sEMG response of paretic extensor carpi radialis better than NMES but did not decrease the cocontraction ratio of flexor carpi radialis.

## Figures and Tables

**Table 1:** Baseline demographics and clinical characteristics.

Characteristic	CCFES (*n* = 25)	NMES (*n* = 25)	*p* value
Age (years)	56.2 ± 12.2	60.4 ± 11.3	0.521
Gender			0.544
Male	18 (72%)	16 (64%)	
Female	7 (18%)	9 (36%)	
Course of disease (days since stroke)	43.9 ± 33.4	46.3 ± 33.1	0.662
Type of stroke			0.382
Ischemic	17 (68%)	14 (56%)	
Hemorrhagic	8 (32%)	11 (44%)	
Hemisphere affected			0.571
Left	11 (44%)	13 (52%)	
Right	14 (56%)	12 (48%)	
Brunnstrom recovery stage			
I-III (hand)	18 (72%)	23 (92%)	
IV (hand)	7 (28%)	2 (8%)	0.066
I-III (upper limb)	20 (80%)	23 (92%)	
IV (upper limb)	5 (20%)	2 (8%)	0.22
ARAT	7.36 ± 12.67	3.44 ± 9.59	0.108
FMA of upper limb	19.52 ± 14.39	14.08 ± 10.35	0.091
Barthel Index	51.20 ± 20.93	46.40 ± 16.80	0.376
RMS of paretic extensor carpi radialis	0.13 ± 0.14	0.12 ± 0.15	0.648
CCR of paretic flexor carpi radialis	0.44 ± 0.18	0.46 ± 0.22	0.673

Data are presented with mean ± SD or numbers (%).

**Table 2:** Changes from baseline to end of intervention in functional assessments and sEMG.

	CCFES (*n* = 25) (mean ± SD)	NMES (*n* = 25) (mean ± SD)	Difference between groups (mean, 95% CI)	*p* value
ARAT	8.92 ± 12.16^^^	3.48 ± 10.44	5.44 (-1.0, 11.89)	0.096
FMA-UE	7.8 ± 7.27^^^	6.04 ± 8.19^^^	1.76 (-2.65, 6.17)	0.43
Barthel Index	11.80 ± 12.90^^^	9.80 ± 10.25^^^	2 (-4.63, 8.63)	0.553
RMS of paretic extensor carpi radialis	0.09 ± 0.16^^^	0.00 ± 0.09	0.09 (0.01, 0.16)^∗^	0.026
CCR of paretic flexor carpi radialis	−0.02 ± 0.179	0.02 ± 0.21	-0.04 (-0.07, 0.15)	0.491

^The difference between end of treatment and baseline is statistically significant, *p* < 0.05. ^∗^The difference between the CCFES and NMES groups is statistically significant.

## Data Availability

The demographic data and data of outcome assessment used to support the findings of this study are available from the corresponding author upon request.
